# Congenital Temporomandibular Joint Ankylosis: Investigating Potential Genetic Etiologies with Whole Exome Sequencing

**DOI:** 10.3390/jcm15041403

**Published:** 2026-02-11

**Authors:** Bożena Anna Marszałek-Kruk, Krzysztof Dowgierd, Mateusz Lejawa, Małgorzata Kulesa-Mrowiecka, Wojciech Wolański, Andrzej Myśliwiec, Anna Lipowicz

**Affiliations:** 1 Department of Genetics, Wroclaw University of Environmental and Life Sciences, 51-631 Wroclaw, Poland; 2 Head and Neck Surgery Clinic for Children and Young Adults, Department of Clinical Pediatrics, Collegium Medicum, University of Warmia and Mazury, 10-561 Olsztyn, Poland; krzysztofdowgierd@wssd.olsztyn.pl; 3 Department of Pharmacology, Faculty of Medical Sciences in Zabrze, Medical University of Silesia, 40-055 Katowice, Poland; mateuszlejawa@gmail.com; 4 Department of Rehabilitation in Internal Diseases, Faculty of Health Sciences, Jagiellonian University Medical College, 31-126 Krakow, Poland; drkulesa@gmail.com; 5 Department of Biomechatronics, Faculty of Biomedical Engineering, Silesian University of Technology, 41-800 Zabrze, Poland; wojciech.wolanski@polsl.pl; 6 Laboratory of Physiotherapy and Physioprevention, Institute of Physiotherapy and Health Sciences, Academy of Physical Education in Katowice, 40-065 Katowice, Poland; a.mysliwiec@awf.katowice.pl; 7 Department of Anthropology, Faculty of Biology and Animal Science, Wroclaw University of Environmental and Life Sciences, 50-375 Wroclaw, Poland; anna.lipowicz@upwr.edu.pl

**Keywords:** ankylosis, Treacher Collins syndrome, Miller syndrome, TCOF1, POLR1B, DHODH

## Abstract

**Background**: Ankylosis of the temporomandibular joint (TMJ) is a rare developmental disorder that involves fibrous or bony fusion within the joint. It is a severe structural and functional disorder. Typically, the phenotype manifests as joint immobilization and results in facial deformity and trismus. To date, ankylosis is rarely diagnosed as congenital and its occurrence mechanism has not been thoroughly understood. We observed a female patient who as a newborn showed slight facial asymmetry and impaired mandibular retraction. In addition, non-uniform occlusal fissures were noted; the lower part of the left earlobe was slightly smaller than the right earlobe. The aim of the work was the identification of pathogenic variants in the genome related to ankylosis. Ankylosis has no known causative gene yet; thus, Whole Exome Sequencing (WES) was performed. **Materials and Methods**: We observed a female patient with facial asymmetry and impaired mandibular retraction from birth. No phenotypic abnormalities were noted on the head or elsewhere on the body. A diagnostic computed tomography (CT) scan of the head performed at five months of age led to the diagnosis of congenital zygomatic-coronoid ankylosis. Genomic DNA samples were subjected to WES. Library preparation was carried out using the Twist Library Preparation EF Kit 2.0, followed by target enrichment with the Twist Exome 2.0 Plus Comprehensive Exome. Sequencing reads were aligned to the human reference genome (GRCh38), and variant calling was performed using standard bioinformatics workflows. Variants were subsequently filtered, annotated, and interpreted using VariantStudio. Assessment of variant pathogenicity was primarily based on comparisons with public databases, including ClinVar and VarSome, and was supported by in silico prediction tools such as SIFT and PolyPhen-2. **Results**: In genes responsible for disorders of the I and II pharyngeal arches, three pathogenic variants were identified: in the genes TCOF1 and POLR1B, responsible for the development of Treacher Collins syndrome (TCS), and one in the DHODH gene, responsible for Miller syndrome. Additionally, in genes that have not been linked so far with rare facial disorders, 42 variants were identified, of which 8 are listed as pathogenic. We present the first described patient with congenital ankylosis, who, although showing no phenotypic features of these syndromes, has identified pathogenic variants in genes responsible for craniofacial dysostosis. **Conclusions**: Variants in TCOF1, POLR1B and DHODH may represent candidate genetic factors associated with susceptibility to ankylosis. WES analysis is an appropriate method in the case of patients with congenital diseases with unknown genetic origin. In this study we provide a comprehensive list of all identified pathogenic variants. This might be useful for scientists searching for the genetic background of skeletal system issues, one of which could be bone and fibrous tissue remodeling.

## 1. Introduction

Ankylosis of the temporomandibular joint (TMJ) is characterized by fibrous or bony fusion within the joint. It represents a severe structural and functional disorder of the face. Clinically, the phenotype manifests as joint immobilization, and results in facial deformity and trismus, which often progress over time. The disorder also causes disturbances in the biomechanics of chewing, swallowing, speech articulation and tongue function. Additionally, ankylosis may reduce airway space, leading to bronchial and pulmonary hypoplasia, and disrupt normal tooth development, leading to malocclusion premature tooth loss and obstructive sleep apnea syndrome (OSAS). These symptoms may subsequently contribute to significant psychosocial problems [1].

Adhesions may present as “true” ankylosis (intra-articular, involving the joint connecting the mandible to the temporal bone), “pseudo” ankylosis (extra-articular, involving structures outside the joint, such as fusion of the coronoid process of the mandible with the zygomatic bone, as observed in zygomatic-coronoid ankylosis), or a mixed form. In cases of acquired ankyloses, in both children and adults, joint stiffness may result from perinatal trauma, post-traumatic injury, infection within the TJM, or postoperative complications [2].

In its congenital form, TMJ ankylosis (ORPHA: 210576) is a rare developmental disorder. It was first described by Burket in 1936 [3]. The frequency of congenital ankylosis and its etiology have yet to be described in the literature. To date, congenital ankylosis has not been reported in patients with syndromes associated with abnormalities of the first and second pharyngeal arches arising during embryonic development (e.g., Treacher Collins syndrome (TCS), Nager, Pierre Robin, or Miller syndromes) [4]. In patients with these defects, abnormal cell differentiation or impaired cell migration occurs during the fetal period, and is characterized by a specific postnatal phenotype (including underdevelopment of the mandible and zygomatic-jaw complex, abnormalities of the external ear, malformation of the ossicles, cleft lip and/or palate and lower eyelid coloboma).

Treacher Collins syndrome is caused by pathogenic variants found in one of four different genes: TCOF1, POLR1D, POLR1C and POLR1B, located on chromosomes 5, 13, 6 and 2, respectively [4]. The typical phenotype of patients with TCS syndrome includes downward-set eyes, underdevelopment of the zygomatic bone and mandible, and a cleft lip and/or palate. TCOF1 is the principal gene associated with Treacher Collins syndrome, with pathogenic variants identified in approximately 90–95% of affected individuals. Variants in the POLR1D, POLR1C and POLR1B genes account for a minority of TCS cases: POLR1D–6%; POLR1C–1.2%; POLR1B–1.3%.

Miller syndrome is a disorder caused by pathogenic variants in the DHODH gene at locus 16q22.2. To date, 23 pathogenic variants in the DHODH gene have been identified in patients with Miller syndrome. Most of these variants result in single amino acid substitutions with dihydroorotate dehydrogenase, likely impairing the ability of enzymes to function normally [5]. Patients with Miller syndrome are typically characterized by hypoplasia, micrognathia, microstomia, cleft lip and/or palate and limb anomalies.

## 2. Materials and Methods

We observed a female patient born at 40 weeks of gestation, the second child of healthy parents. She was delivered vaginally in October 2015, without complications. Her physical condition as a newborn was rated as 10 points on the Apgar scale. Birth weight was 3470 g, crown rump length 36 cm, head circumference 33.5 cm, and chest circumference 34 cm. After birth, the newborn showed mild facial asymmetry and impaired mandibular retraction. The first diagnosis from a specialist in the field of craniofacial defects was after 5 days, assessed as hemifacial microsomia with limited mouth opening to 3 mm. In addition, non-uniform occlusal fissures were noted (larger on the right side than on the left side), and the lower part of the left earlobe was slightly smaller than the right earlobe [6]. There were no phenotypic changes in the orbital area, cleft lip or palate, micrognathia, auricles, nor limb defects. There were no phenotypic abnormalities on the head or the rest of the body. No relevant conditions were reported for the parents, an older sister and close relatives.

**Figure 1:**
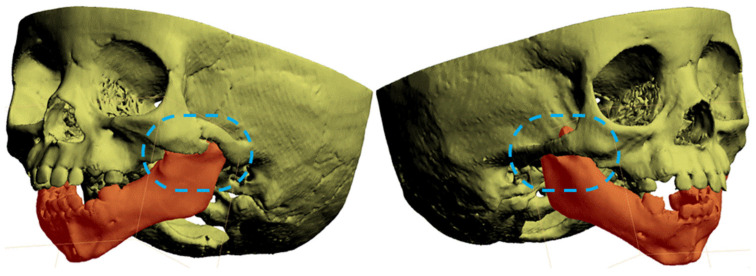
3D model of the patient’s head with diagnosed congenital zygomatic-coronoid ankylosis. The severe syngnathia is visible on the left side (left side of the head). The right side of the head (mild pathological changes) is visible on the right (areas in blue dotted lines indicate visible difference in ankylosis severity between left and right side).

A diagnostic computed tomography (CT) scan of the head performed at five months of age led to a diagnosis of congenital zygomatic-coronoid ankylosis, consisting of adhesions of the coronoid process of the mandible and zygomatic arch on the left side of the face (Figure 1). Before the age of eight years, the patient underwent a series of surgical procedures to remove the mandibular adhesion. Due to recurrent ankylosis, these procedures were repeated, and finally resection of the coronoid processes and articular processes and prosthetics of the temporomandibular joints were performed on both sides of the face. Throughout the treatment period, the patient continuously received close physiotherapeutic care [6].

Physiotherapeutic care is essential to maximize postoperative outcomes and minimize reoccurrence of ankylosis. In this particular case, due to stomatognathic physiotherapy, mouth opening increased from 3 mm to 30 mm with significant improvement for food intake, articulation and psychosocial aspects of the developing child. Immediate physiotherapy after surgery—coronoidectomy to release congenital TMJ ankylosis—has to be fast and efficient and to restore biomechanical and physiological functions of the stomatognathic system [7].

Although provided care, the patient received the following procedures to treat zygomatic arch ankylosis:

- Age 2—surgery to release ankylosis of the mandibular coronoid process with zygomatic arch on the left side. After the procedure, there was a recurrence of the ankylosis (reankylosis);
- Age 3—removal of the reankylosis obstruction of the left temporomandibular joint: patient intubated with a fibreoptic scope, intubation extremely difficult;
- Age 7—mandibular osteotomy with placement of distractors bilaterally, removal of bilateral mandibular distractors;
- Age 8—bilateral temporomandibular joint prosthesis.

The patient and her treatment has been previously reported [6,8,9,10].

Due to recurrent soft tissue and bone tissue adhesions, a decision was made to perform genetic testing. Blood was drawn from the patient, and genomic DNA was isolated using a Sherlock™ DNA extraction kit. A PCR reaction amplified the DNA and the sample underwent WES (Whole Exome Sequencing) analysis.

### 2.1. Genetic Analyses

Genomic DNA samples were subjected to WES. Library preparation was performed using the Twist Library Preparation EF Kit 2.0, followed by target enrichment with the Twist Exome 2.0 Plus Comprehensive Exome, in accordance with the manufacturer’s instructions. Genomic DNA was randomly fragmented, and sequencing adapters were ligated to both ends of the DNA fragments. Pooled libraries were quantified using quantitative PCR.

Sequencing was performed on an Illumina NovaSeq 6000 using paired-end reads (2 × 151 bp). Raw image data were processed using Illumina control software, and base calling was performed using Real-Time Analysis (RTA). The resulting data were converted to FASTQ format, including Phred quality scores, using bcl2fastq.

### 2.2. Bioinformatic Analysis

Quality assessment of raw sequencing reads was performed using FastQC (v0.11.8). Adapter sequences were removed with Cutadapt (v2.8). Filtered reads were aligned to the human reference genome GRCh38 (hg38) using the BWA-MEM algorithm (v0.7.15).

Variant calling was performed using the HaplotypeCaller module in the Genome Analysis Toolkit (GATK v4.2.2). Variant filtering followed GATK best-practice recommendations, using the VariantFiltration tool. Both variants passing quality filters (PASS) and variants failing these filters were retained in the VCF files to allow downstream evaluation of variant quality.

Sequencing achieved high coverage of the targeted regions, with a mean depth exceeding 150× per sample and more than 98% of target bases covered at a minimum depth of 20×. Detected variants included single-nucleotide polymorphisms (SNPs) and short insertions and deletions (indels), and were represented in normalized VCF format. Final output files (FASTQ, BAM, and VCF) were visually inspected using the Integrative Genomics Viewer (IGV) to assess read alignment quality, coverage, and variant calls.

Genomic variants were further filtered and annotated using VariantStudio (Illumina, Inc. (San Diego, CA, USA). Classification of variants as pathogenic or likely pathogenic was based primarily on comparisons with public databases such as ClinVar and VarSome, supplemented by in silico predictions from tools including SIFT [11] and PolyPhen-2 [12].

## 3. Results

### WES Analysis

Genomic variants were filtered and annotated. Variants with frequencies higher than 1% reported in the available databases were excluded, as well as synonymous and intronic variants outside of the flanking regions. Missense variants were analyzed with algorithms using functional annotation, including SIFT (https://sift.bii.a-star.edu.sg) (accessed on 14 October 2025) and PolyPhen-2 (http://genetics.bwh.harvard.edu/pph2/) (accessed on 14 October 2025).

The functional effects of the missense variants listed in Table 1 were assessed in silico using two widely used pathogenicity prediction tools: SIFT and PolyPhen-2. SIFT predicts whether an amino acid substitution is tolerated or deleterious based on evolutionary conservation and physicochemical similarity; variants with SIFT scores ≤0.05 were considered deleterious, while scores >0.05 were considered tolerated. PolyPhen-2 predicts the potential impact of amino acid substitutions on protein structure and function using sequence- and structure-based features (including domain annotations such as Pfam and, when available, 3D structural information from PDB). PolyPhen-2 scores range from 0.0 (benign) to 1.0 (deleterious) and were interpreted as benign (≤0.446), possibly damaging (>0.446–≤0.908), or probably damaging (>0.908) (PolyPhen-2 HumVar model). These predictions were used to support the putative functional impact of the variants reported in Table 1.

The WES analysis assayed for pathogenic gene variants, which were summarized into two key groups. The first included genes responsible for known rare genetic diseases associated with the I and II pharyngeal arches, such as Treacher Collins syndrome (OMIM 154500), Nager syndrome (OMIM 154400), Miller syndrome (also known as Genée–Wiedemann syndrome, Wildervanck–Smith syndrome or postaxial acrofacial dysostosis) (OMIM 263750) and Pierre Robin syndrome (OMIM 261800). The suspected genes for the aforementioned diseases are TCOF1, POLR1D, POLR1C, POLR1B, SF3B4, DHODH and EFTUD2. The second group of target genes was a panel of pathogenic or likely pathogenic variants.

In the first panel of target genes, three pathogenic variants were identified in the patient: one in TCOF1, one in POLR1B associated with Treacher Collins syndrome, and one pathogenic variant in the DHODH gene, which is responsible for Miller syndrome.

POLR1B c.998C>T is described in the Catalogue of Somatic Mutation In Cancer (COSMIC, ID:COSM4001143). Expression of POLR1B is mainly associated with soft tissue. In the NCBI database, the variant is also registered as a Small Nucleotide Polymorphism (SNP), rs1545133.

TCOF1 c.3527C>G is listed in the ClinVar database under VCV000130571.1, rs1136103. It has been observed in association with one case of Treacher Collins syndrome, with benign or uncertain significance. This may have been spuriously associated with a single case. However, it is stated that there is sufficient evidence for dosage pathogenicity. This same finding was confirmed in the Varsome database https://varsome.com/variant/hg38/rs1136103 (accessed on 15 October 2025).

DHODH c.19A>C (COSMIC ID: COSM1379664) is related to soft tissue, and has been reported in six cases of patients with gastrointestinal stromal tumors, as well as in association with one benign case of Miller syndrome. Listed in Clinvar as VCV000128895.2; rs3213422.

Suspected pathogenicity was confirmed by comparing variants with databases including Varsome (https://varsome.com) (accessed on 16 October 2025), ClinVar (https://www.ncbi.nlm.nih.gov/clinvar/) (accessed on 17 October 2025), and others. All three cases of missense variants in genes related to rare facial syndromes were confirmed using web programs https://mutalyzer.nl/normalizer/ (accessed on 5 November 2025) and https://www.mutationtaster.org (accessed on 6 November 2025). None of the previous case descriptions associated with these variants had reported ankylosis.

In summary, the panel of assayed genes known to be associated with dysmorphological rare syndromes affecting facial development used here was not related to previously reported ankylosis in any previously identified patients. However, two variants in TCOF1 and DHODH, which we observed in the patient, have previously been identified in patients with Treacher Collins and Miller syndromes.

In the second panel of genes, excluding those suspected to be associated with rare facial disorders, 42 variants were identified, of which 8 are listed as pathogenic or likely pathogenic in ClinVar. These findings are rather incidental, not related to craniofacial development. The closest connection to craniofacial development may have variants in gene OTOG c.2500C>T, which could cause deafness; COL4A3 c.4421T>C, which could cause Alport syndrome responsible for kidney disease, but occurring with hearing loss; and SBDS c.258+2T>C, responsible for Shwachman–Diamond syndrome, affecting bones. The rest of the listed variants and their genes could be responsible for non-facial disease: dysfibrinogenemia, hypofibrinogenemia and thrombophilia, tracheoesophageal fistula, and spastic paraplegia. A further 34 instances of variation observed in the patient, which were not previously published in COSMIC or ClinVar, were analyzed in other databases. These 42 identified variants were located on different chromosomes across the genome. On chromosomes 8, 12, 13, 17, 18, 20, 21, 22, no variants were found. There was no clear correlation between the number of variants and their position on the chromosomes; however, the variant occurrence was the highest on chromosomes 1, 2 and 3 (respectively 4, 6, and 5 cases). Of 27 unpublished variants, 20 are frameshift mutations, and 5 are loss-of-function variants (Table 2).

## 4. Discussion

WES analysis was performed for the patient, who had been diagnosed with and surgically treated for congenital idiopathic ankylosis. This analysis revealed, among other findings, concomitant pathological changes in the TCOF1, POLR1B and DHODH genes. These pathogenic variants are associated with defects of the I and II pharyngeal arches, underlying RNA polymerase transcription disorders (ribosomopathies). Mutations in the TCOF1 and POLR1B genes are responsible for the development of Treacher Collins syndrome (TCS), while mutations in the DHODH gene are responsible for Miller syndrome (MS). To date, no cases of TCS or MS reported in the literature have involved temporomandibular joint ankylosis, either affecting the articular process (true ankylosis—intra-articular) or the coronoid process (pseudo ankylosis—extra-articular).

To date, congenital ankylosis has not been genetically described or characterized in patients with syndromes associated with abnormalities of the first and second pharyngeal arches arising during embryonic development (e.g., Treacher Collins syndrome (TCS), Nager, Pierre Robin, or Miller syndromes). Rosa et al. primarily described Pierre Robin Sequence (PRS) in terms of clinical features [13]. Genetics aspects associated with the triad of micrognathia, glossoptosis, and airway obstruction, often associated with cleft palate, but without indication of ankylosis of TMJ, were discussed by Karempelis et al. [14]. Kulesa-Mrowiecka et al. reported the rehabilitation of a child with Pierre Robin Sequence accompanied with TMJ ankylosis, which included manual therapy of the temporomandibular joint, feeding training, and polysensory, proprioceptive exercises [15].

**Figure 2:**
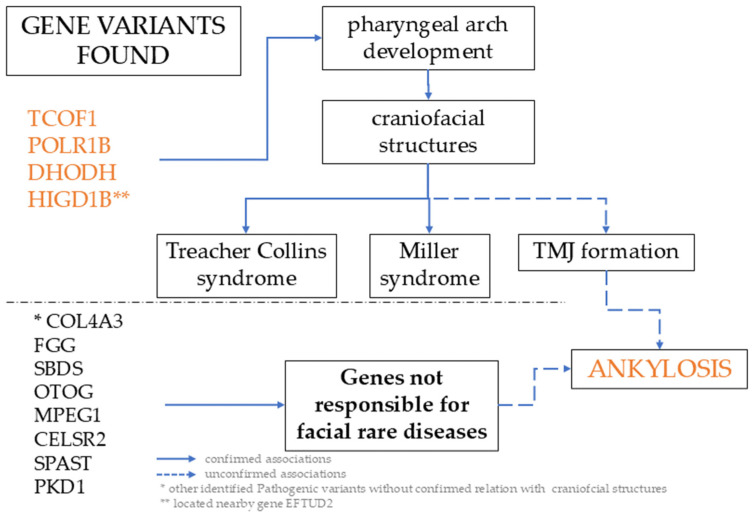
Conceptual pathway of the study. Three identified pathogenic variants are located in the same gene, which is responsible for well genetically described syndromes. The genetic basis of idiopathic TMJ ankylosis is unknown. Orange fond indicate new finding: relation between genes which pay role in pharyngeal arch development and ankylosis.

The pathogenesis of congenital ankylosis remains poorly understood. Its genetic determinants are unknown, and its origin has not been linked to any recognized orofacial–mandibular syndrome. The patient described is an exceptionally rare case, with simultaneous presence of three pathogenic variants in three different genes, each of which affects the development of the I and II pharyngeal arches. Two of these variants independently cause TCS, and the third causes Miller syndrome, another form of facial dysostosis (Figure 2). Despite the simultaneous presence of these three pathogenic variants, the patient exhibited no phenotypic abnormalities at birth, or during early childhood, that would suggest the underlying existence of dysmorphic genetic variants. We speculate that the absence of TCS or MS phenotypic features, despite the presence of responsible variants, may be explained by variable expressivity or incomplete penetrance of the variants.

The use of WES method enabled the identification of a potential genetic basis for isolated, idiopathic congenital ankylosis of the craniofacial region. The pathogenic variants listed in the second panel of genes have not been previously identified as associated with syndromes of the I and II pharyngeal arches. Ankylosis, as a condition that is rarely diagnosed as congenital, currently has no known causative pathogenic variants. These findings raise several questions that could be addressed through further genomic studies involving both individuals with congenital ankylosis and those with orofacial dysostoses, as well as their families.

## 5. Conclusions

The TCOF1, POLR1B and DHODH genes may potentially play a common role in disorders of facial development and ankylosis. WES analysis is an appropriate method for investigating patients with congenital diseases of unknown genetic origin. In this study we provide a comprehensive list of all identified pathogenic variants. This might be useful for scientists researching the genetic background of skeletal system issues, one of which could be bone and fibrous tissue remodeling. Early and prompt diagnosis of ankylosis could contribute to the use of effective and more appropriate surgical and physiotherapeutic treatment.

## Figures and Tables

**Table 1:** Results of the WES analysis: missense variant scores and predictions.

Pathogenic Variant and Effect Result from SHIFT and PolyPhen-2: Scores and Effect Predictions							
Variant ID	Gene	Reference SNP	Consequence	Sift		PolyPhen	
				Prediction	Score	Prediction	Score
2:112551896:T	POLR1B	rs1545133	missense_variant	tolerated	0.46	benign	0.0
5:150392717:G	TCOF1	rs1136103	missense_variant	deleterious	0.01	possibly damaging	0.88
16:72008783:C	DHODH	rs3213422	missense_variant, splice_region_variant	tolerated	0.19	benign	0.005
17:44850353:A	HIGD1B	rs1071682	missense_variant	tolerated	0.21	benign	0.0
2:227307878:C	COL4A3	rs200302125	missense_variant	deleterious	0.0	probably damaging	1.0
4:154611883:C	FGG	rs148685782	missense_variant	tolerated	0.33	benign	0.01
7:66994210:G	SBDS	rs113993993	splice_donor_variant				
11:17574890:T	OTOG	rs554847663	stop_gained				
11:59211653:T	MPEG1	rs200420254	missense_variant	deleterious	0.0	possibly damaging	0.785
1:109252937:G	CELSR2	rs1223692503	missense_variant	deleterious	0.0	probably damaging	1.0
2:32154383:G	SPAST	rs1259072587	missense_variant	deleterious	0.02	possibly damaging	0.46
16:2118021:A	PKD1	rs199476099	missense_variant	deleterious	0.04	benign	0.245

**Table 2:** Results of the WES analysis, detailing pathogenic variants found in a patient with ankylosis. The top three rows identify genes responsible for rare facial diseases in characterized syndromes. The bottom eight rows detail pathogenic or likely pathogenic variants found in groups of genes not suspected to be associated with known rare genetic syndromes.

**Pathogenic Variants Identified in Genes Known to be Responsible for Deformations in First and Second Facial Arches**					
**Variant ID**	**Gene**	**Exon**	**HGVSC**	**HGVSP**	**Zygosity**
2:112551896:T	POLR1B	7/16	c.998C>T	p.(Ser333Leu)	Homozygous
5:150392717:G	TCOF1	22/27	c.3527C>G	p.(Pro1176Arg)	Heterozygous
16:72008783:C	DHODH	1/9	c.19A>C	p.(Lys7Gln)	Homozygous
17:44850353:A	HIGD1B	4/4	c.257G>A	p.(Ser86Asn)	Heterozygous
**Pathogenic Variants Identified in Genes Not Known to be Responsible for Rare Facial Diseases**					
**Variant ID**	**Gene**	**Exon**	**HGVSC**	**HGVSP**	**Zygosity**
2:227307878:C	COL4A3	48/52	c.4421T>C	p.(Leu1474Pro)	Heterozygous
4:154611883:C	FGG	4/9	c.323C>G	p.(Ala108Gly)	Heterozygous
7:66994210:G	SBDS		c.258+2T>C		Heterozygous
11:17574890:T	OTOG	19/55	c.2500C>T	p.(Gln834Ter)	Heterozygous
11:59211653:T	MPEG1	1/1	c.1213C>A	p.(Pro405Thr)	Heterozygous
1:109252937:G	CELSR2	1/34	c.2858A>G	p.(Asn953Ser)	Heterozygous
2:32154383:G	SPAST	17/17	c.1738A>G	p.(Ile580Val)	Heterozygous
16:2118021:A	PKD1	5/46	c.971G>T	p.(Arg324Leu)	Heterozygous

## Data Availability

The datasets used and/or analyzed during the current study are available from the corresponding author on request.

## Abbreviations

The following abbreviations are used in this manuscript:

COSMIC	Catalogue of Somatic Mutation In Cancer
CT	Computed tomography
MS	Miller syndrome
OSAS	Obstructive Sleep Apnea Syndrome
OMIM	Online Mendelian Inheritance in Man
PCR	Polymerase Chain Reaction
TMJ	Temporomandibular Joint
WES	Whole Exome Sequencing
